# RNA thermometers as temperature-dependent tools for enhanced recombinant protein production in precision fermentation

**DOI:** 10.1186/s12934-026-03143-2

**Published:** 2026-10-13

**Authors:** Christina Peternell, Philipp Noll, Marius Henkel

**Affiliations:** Cellular Agriculture, TUM School of Life Sciences, Technical University of Munich, Gregor-Mendel-St. 4, 85354 Freising, Germany

**Keywords:** Precision fermentation, Thermoregulation, RNA thermometer, Temperature-dependent expression, Regulatory RNA, Inducer-free expression control, Molecular process control, Synthetic biology, Dynamic bioprocess regulation

## Abstract

**Background:**

Precision fermentation is a key technology for the future sustainable production of food proteins, bulk ingredients, and high-value recombinant products. However, process efficiency is often limited by the coupling of biomass formation and recombinant protein synthesis, leading to substantial carbon losses into non-recyclable biomass. Particularly for cost-sensitive bulk products such as food proteins, economically viable production requires highly efficient and dynamically controllable bioprocesses. RNA thermometers (RNATs) offer a promising inducer-free strategy for temperature-dependent control of recombinant protein production and dynamic bioprocess regulation, with the potential to improve carbon efficiency and product yields.

**Results:**

In this study, a fluorescent reporter protein (EmGFP) under the control of a fourU (4U) RNA thermometer was evaluated in *E. coli* BL21 across a temperature range of 25–40 °C. Temperature-dependent regulation enabled maximal recombinant protein production at 35 °C, resulting in a 2.6-fold higher specific productivity compared to the derepressed control. The strongest RNAT-mediated regulatory effect was observed at 35.5 °C, highlighting the relevance of precise temperature control for recombinant protein production. Evaluation of altered carbon partitioning revealed a substantially increased carbon allocation toward product formation, while carbon partitioning into biomass and CO_2_ remained largely unchanged. Furthermore, the product-per-acetate yield increased more than threefold at induction temperature, indicating controllable carbon partitioning and reveals potential for decoupling approaches of biomass formation from recombinant protein production.

**Conclusion:**

This study demonstrates the successful implementation of an RNAT-controlled recombinant expression system enabling temperature-dependent regulation of protein production in *E. coli*. Quantitative analysis revealed controllable carbon partitioning toward recombinant protein synthesis and defined target temperatures for future process induction and switching. In contrast to conventional inducer-based systems, RNAT-mediated regulation enables dynamic control without chemical additives, supporting more resource-efficient bioprocess operation. However, fully exploiting RNAT-based process control will require advanced model-based strategies integrating temperature and feeding regimes, particularly for cost-sensitive precision fermentation processes such as food protein production. Overall, RNATs represent promising molecular control elements for future high-efficiency precision fermentation processes.

**Supplementary Information:**

The online version contains supplementary material available at https://doi.org/10.1186/s12934-026-03143-2.

## Background

The increasing demand for alternative protein sources has intensified interest in microbial production systems. In particular, the economically viable production of bulk food proteins and functional protein additives requires highly efficient bioprocesses to compete with conventional protein sources such as dairy or egg-derived products [1, 2]. Precision fermentation reframes established recombinant protein production technologies toward the sustainable manufacturing of food proteins and other specific target proteins for industrial applications. As a platform technology, it has the potential to increase the independence, robustness, and flexibility of future food supply systems while reducing land requirements compared to traditional agriculture [3]. A recent literature review states that titers > 50 g/L of recombinant protein must be achieved to ensure economically viable production of alternative proteins. To meet these technically challenging titers, production hosts and bioprocesses need to be highly efficient and controllable [2]. Strain development, metabolic engineering, process optimization, and scale-up are mentioned as crucial prerequisites for the success of recombinant alternative proteins from PF. Additionally, these tools support a safe and sustainable future food supply [4–8].

A neglected starting point for increasing the efficiency of bioprocesses is the gradual decoupling of biomass and product formation. In most cases, biomass is a by-product that is difficult to recycle efficiently, and consequently, substrate carbon gets lost. Furthermore, considerable amounts of carbon are potentially redirected into by-products. For instance, in *Escherichia coli*, acetate accumulation above 1–2 g/L is frequently reported as inhibitory to cell growth and heterologous protein production [9–12]. This phenomenon is described as overflow metabolism in prokaryotes or Crabtree effect in yeasts, and has been intensively reviewed [13–18]. Bioprocess control so far is mostly limited to the addition of fresh media in fed-batch processes. As an alternative to induction of recombinant processes with (non-food safe) chemical inducers like isopropyl β-D-1-thiogalactopyranoside (IPTG), constitutive promoter systems are applied. Nevertheless, these systems do not allow separated cultivation phases of biomass and product formation [19–21].

One way to introduce an additional layer of bioprocess control is to use molecular control elements for gene expression, like quorum sensing or auxotrophic mutations, as intensively reviewed by Treinen and colleagues [22]. A simple one-step mechanism without the need for additional signal molecules is to use regulatory RNAs, such as RNA thermometers (RNAT) [22–25]. RNATs were initially discovered in 1989, controlling the bacteriophage lambda *cIII* gene expression [26]. RNATs are temperature-responsive secondary RNA structures (mostly) located in the 5’-untranslated region (5’-UTR). Hoe and Goguen described the expression of virulence factor gene *lcrF* in *Yersinia pestis* and experimentally tested it in *E. coli* already in the early 1990ies comparing expressions at 26 °C and 37 °C [27]. Findings show that LcrF protein appears thermostable at 26 °C and 37 °C however, lower levels of protein were observed for 26 °C. By performing mRNA secondary structure prediction SD sequence is masked by being part of a stem loop with high probability, and increased temperature favors gene expression by destabilizing the conformation [27].

**Fig. 1:**
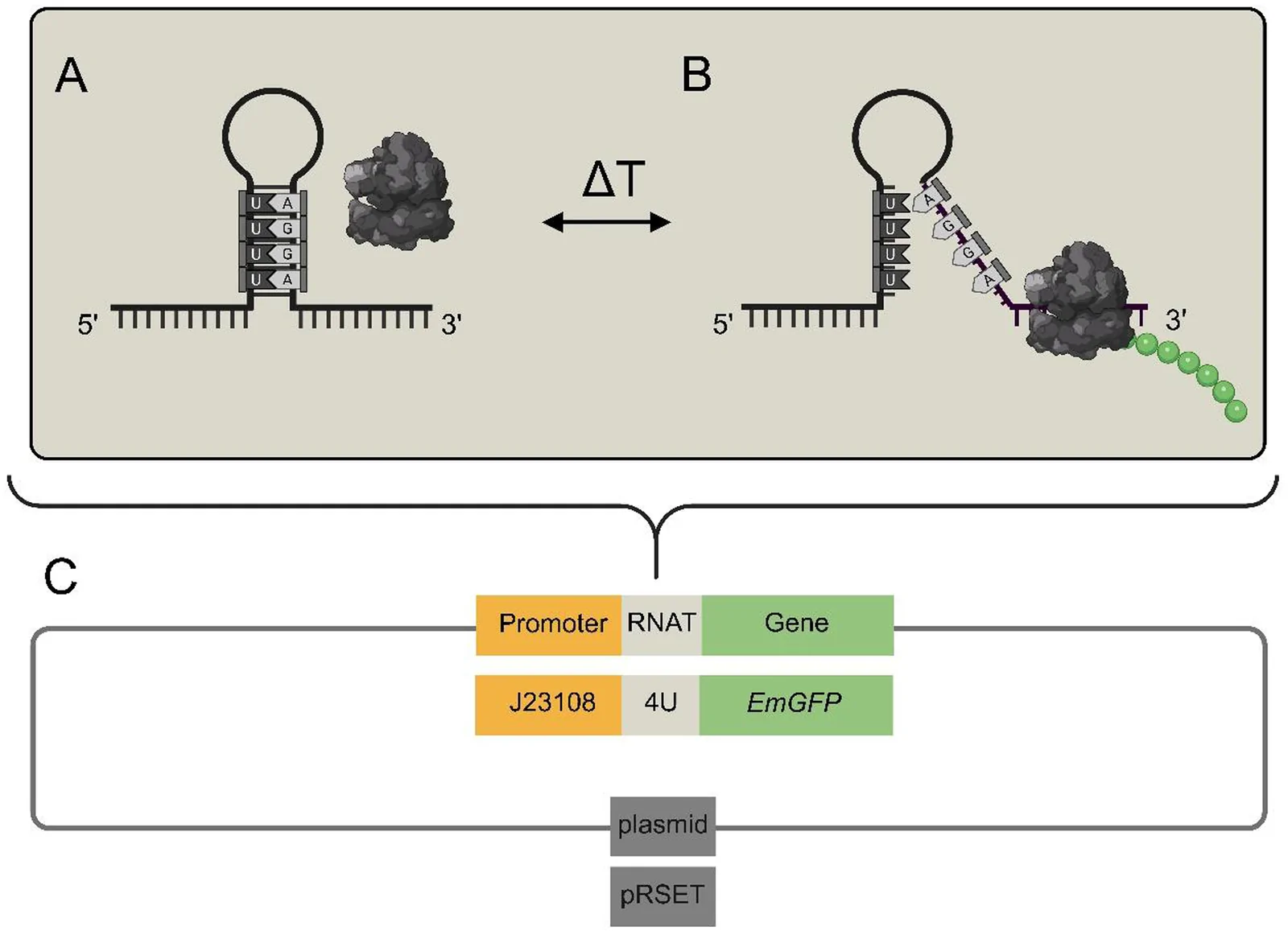
Schematic representation of the fourU (4U) RNA thermometer (RNAT) based expression system. **A**) active regulatory mechanism that masks ribosome binding site (RBS), **B**) temperature-induced conformational change favoring translation by exposing RBS, **C**) exemplary plasmid construct pRSET_J23108_4U_EmGFP. Created in BioRender. Peternell, C. (2026) https://BioRender.com/d82eeg5

Two RNAT families have been reported: the ROSE(-like) RNATs and the fourU (4U) RNATs. ROSE is short for repression of heat shock gene expression and the RNATs within this family are most commonly described in connection with the translational control of heat shock genes e.g. heat shock protein A (*hspA*) in *Bradyrhizobium japonicum* [28, 29]. An exception to this rule poses the expression control of rhamnolipids, a virulence factor of the human pathogen *Pseudomonas aeruginosa* [30, 31]. The second family contains the 4U RNAT, so called because of its characteristic four Uracil motif complementary to the RBS (Shine-Dalgarno, SD sequence: AGGA) within the inhibitory hairpin. 4U RNAT are most commonly associated with virulence gene expression [29, 32–34]. They serve in this regard as a thermosensor that allows the sensing of a host’s temperature upon invasion. This then triggers the expression of virulence genes when such a temperature change (usually from ambient temperature to 37 °C) is sensed. An exception to 4U RNATs regulating virulence is the expression control of a small heat shock gene (*agsA*) from *Salmonella enterica*. A subsection (hairpin 2) of the entire RNAT has been reported to already have a regulatory effect with not more than 45 nucleotides in length [25]. The hairpin 2 of the 4U RNAT regulating *agsA* is used within this work and is abbreviated as “T-resp.”, temperature-responsive construct. As described by Waldminghaus and colleagues, the sequence has been modified to yield a mutant structure with a modulated stability (described as “destabilized” and “derepressed”) by exchanging G14 with an A and C25 with an U. The 4U G14A-C25U mutation is used in this work as a derepressed control and is abbreviated as “control” [25]. RNATs as additional molecular layers, function as dimmer-switches opening up various ways to interfere in a bioprocess and optimize it by using temperature as a physical control variable [24]. Figure 1 schematically demonstrates the temperature-responsive conformational change. At inhibitory temperatures, RNATs (in most cases) mask the ribosome binding site (RBS), preventing translation. Upon a temperature change, the inhibitory structure unfolds and liberates the RBS, allowing ribosome annealing and translation initiation [6, 23, 35, 36].

As another key advantage, RNAT can be used across species since its functionality depends merely on a physical conformational change. Nocker and team presented the functionality of a ROSE element in various species, such as *Bradyrhizobium* sp., *Rhizobium* sp., or *Mesorhizobium* [28]. Another study by Kammerdiener and colleagues observed the transferability of a RNAT from *E. coli* into *P. putida* [37]. Furthermore, protocols for the design and testing of synthetic RNATs with a defined temperature response range are available [38–42]. An advanced approach presented by Jia and team investigated the capability of RNAT combined with quorum sensing regulation for increasing the thermos-robustness of *E. coli* cells [43]. Although RNATs have been extensively studied with respect to their temperature response range, occurrence across different organisms, and structural folding mechanisms, their application as regulatory elements for recombinant protein production remains limited. To our knowledge, only previous work by Noll and colleagues investigated the use of RNATs for a biosurfactant production of rhamnolipid, revealing initial steps toward the implementation in biotechnological processes [31, 44].

This study evaluates the general capabilities of RNATs as an induction element for recombinant protein expression. A reference system was established in the model strain *E. coli* BL21 and designed as a modular construct, making it suitable for different production strains and target proteins. These systems are of particular interest in the context of precision fermentation, where efficient and controllable expression of food-relevant proteins is required. The induction strength of the heterologous RNATs (4Uwt, T-resp.) controlling the translation of a constitutively transcribed reporter gene (Emerald green fluorescent protein, EmGFP) is quantified and compared to a previously characterized control sequence (4U derepressed, mutation G14A-C25U, control) [33]. To do so, time courses of 24 h cultivation time (biomass, substrate, product, and by-product) are recorded, and growth, production rates, and yield coefficients over tested temperature range are quantified. Cultivations in shake flasks were conducted at physiological temperatures between 25 –40 °C. The gathered data is used for a temperature model that describes temperature-dependent average specific EmGFP production. To evaluate altered carbon partitioning in both the T-resp. and the control system, biomass, product, and side-product formation (acetate) per substrate are evaluated and presented as relative fractions.

## Methods

### Microorganism and plasmids

*E. coli* cells were purchased from formerly Stratagene CA, La Jolla, CA, USA (*E. coli* BL21, production host) and Thermo Scientific Inc., Waltham, MA, USA (*E. coli* DH5α, plasmid propagation and storage). RNA thermometer (RNAT) expression system was constructed using commercial plasmid pRSET-EmGFP (V35320, Invitrogen by Thermo Scientific, Waltham, MA, USA) as a backbone. Translation of EmGFP reporter gene is under the control of fourU wildtype (4Uwt, “T-resp.”) or fourU derepressed mutant (4Udest, mutation G14A-C25U, “control”) native to *Salmonella*, presented in Table 1 and in the supplementary information, Figure S1, combined with a constitutive promoter.

### Plasmid assembly

Plasmid assembly was performed according to Peternell et al., 2026 and is schematically illustrated in Fig. 1 [45]. Plasmid maps are provided in the supplementary information, Figure S2. The constitutive promoter, RNAT, and the start sequence of the gene of interest Emerald green fluorescent proteins (EmGFP, 5‘untranslated region (5’-UTR) up to codon nine of EmGFP) was obtained in a pEX-A128 plasmid from Eurofins Genomics Germany GmbH Hamburg, Germany with an ampicillin selection marker. The segment was amplified, digested (#R0581S *BseRI* and *#*R0655S *PciI*, New England Biolabs GmbH, Frankfurt am Main, Germany) and ligated (*T4 DNA Ligase* EL0014, Thermo Scientific Inc., Waltham, MA, USA) into the working backbone pRSET-EmGFP. The promoter J23108 of the Anderson promoter family [46] was employed in the production plasmids. Plasmids were transferred by heat shock into chemically competent *E. coli* DH5α (Thermo Scientific Inc.) for storage and into chemically competent *E. coli* BL21 as a working strain. *E. coli* BL21 without carrying a plasmid (no plasmid construct, “NP”) was cultivated as a control without suffering from metabolic burden. Transformants were stored at -80 °C in 20% (v/v) glycerol stocks.

**Table 1:** Sequences of 4U RNA thermometers from *Salmonella* applied in this study

RNAT	Sequence 5’ → 3’	References
FourU wildtype (4Uwt, “T-resp.”)	GUUGAACUUUU**G**AAUAGUGAUU**C**AGGAGGUUAAUG	[25, 32–34]
FourU derepressed, mutant G14A-C25U (4Udest, “control”)	GUUGAACUUUU**A**AAUAGUGAUU**U**AGGAGGUUAAUG	[6, 33, 44]

Point mutation is highlighted in bold

### Chemicals and substances

All chemicals used in this study were obtained from Carl Roth GmbH & Co. KG (Karlsruhe, Germany), VWR International GmbH (Darmstadt, Germany), Bio-Rad Laboratories, Inc. (Hercules, CA, USA), and Merck KGaA (Darmstadt, Germany), unless stated otherwise.

### Media preparation

Cultivation media and solutions were prepared according to Bertani, 1951; Dulbecco and Vogt, 1954; Henkel et al., 2015 as described previously by Peternell et al., 2026 [45, 47–49].

### Cultivation in shake flasks

Shake flask cultivations were conducted at 160 rpm in a shaking incubator (Minitron, Infors HT, Bottmingen, Switzerland) with 25 mm of shaking diameter. 25 mL of LB media prepared in a 250 mL baffled shake flask were inoculated with 25 µL glycerol stock of *E. coli* BL21 NP, *E. coli* BL21 T-resp., or *E. coli* BL21 control. Pre-culture was incubated for 10 h at 37 °C. 100 mL of M9 production media with 10 g/L glucose was prepared in a 1000 mL baffled shake flask and inoculated to an optical density (OD_600_) of 0.1 with the pre-culture. M9 production cultivation was conducted at different constant temperatures between 25 and 40 °C [25.0; 30.0; 32.5; 35.0; 37.5; 40.0] over 24 h.

### Sampling and processing

Samples (2 mL) were drawn every two hours, unless stated otherwise, over 24 h. Biomass concentration determination was performed via turbidity measurement at a wavelength of 600 nm (Ultrospec™ 10 Cell Density Meter, Harvard Bioscience Inc., Holliston, MA, USA) using factor 0.334 ± 0.003 g/L for OD_600_ 1.0 according to Peternell et al., 2026 [45]. 200 µL of cell suspension were used for EmGFP quantification [mg/L] via relative fluorescence units (RFU) [-] determination. 1 mL of cell suspension was centrifuged at 16,000 × g for 6 min at 4 °C (Rotina 380R, Hettich GmbH & Co.KG Tuttlingen, Germany), storing supernatant and cell pellet in separate tubes at -20 °C until subsequent assays.

### Glucose quantification assay

Glucose concentration [g/L] of cell-free supernatants was analyzed using a D-glucose assay kit (K_GLUC D-Glucose Assay Kit, GOPOD Format, 700004297, Megazyme Ltd., Wicklow, Ireland) according to the manufacturer’s protocol adapted as a microtiter plate approach (96-well plate, 655.101, Greiner Bio-One GmbH, Frickenhausen, Germany), decreasing the volume of the cuvette approach to 1/12.5. Absorbance was measured at 510 nm, with 10 flashes and a settle time of 50 ms per well in a microplate reader (Tecan Spark reader, Tecan Spark reader, Tecan Trading AG, Männedorf, Switzerland) with SparkControl Magellan and MethodEditor software (Tecan Group Ltd., Männedorf, Switzerland). A glucose standard curve (R² > 0.99) was generated using a dilution series (0.0, 0.2, 0.4, 0.6, 0.8, 1.0 g/L glucose) and corresponding absorbance values (A_510nm_). Samples were analyzed in technical duplicates of biological triplicates.

### Acetate quantification assay

Acetate concentration [g/L] of cell-free supernatants was analyzed using an acetic acid assay kit (Enzytec™ Liquid Acetic acid, E8226, R-Biopharm AG, Darmstadt, Germany) according to the manufacturer’s protocol adapted as a micro titer plate approach (96-well plate, 655.101, Greiner Bio-One GmbH, Frickenhausen, Germany), decreasing the volume of the cuvette approach to 1/10. Absorbance was measured at 340 nm, with 10 flashes and a settle time of 50 ms per well in a microplate reader (Tecan Spark reader, Tecan Spark reader, Tecan Trading AG, Männedorf, Switzerland) with SparkControl Magellan and MethodEditor software (Tecan Group Ltd., Männedorf, Switzerland). An acetic acid standard curve (R² > 0.99) was generated using a dilution series (0.02, 0.1, 0.3, 1.3 g/L acetate) and corresponding absorbance values (A_340nm_). Samples were analyzed in technical duplicates of biological triplicates.

### Fluorescence measurement and EmGFP quantification [mg/L]

EmGFP concentration [mg/L] was quantified via monitoring of relative fluorescence units (RFU) [-] in a microplate reader (Tecan Spark reader, Tecan Trading AG, Männedorf, Switzerland) with SparkControl Magellan 3.0 and MethodEditor software (Tecan Group Ltd., Männedorf, Switzerland) using the setting *eGFP* from the fluorophore dropdown list). 200 µL of cell suspension sample was measured in a black 96-well plate (655.076, Greiner Bio-One GmbH, Frickenhausen, Germany) with excitation at 487 nm, emission at 540 nm, fixed gain set to 60, and a Z - position of 20,000 μm. Samples were analyzed in technical duplicates of biological triplicates.

Conversion of measured RFU [-] of cell suspension into absolute EmGFP concentration [mg/L] was performed as described by Peternell et al., 2026 [45]. Absolute EmGFP [mg/L] was calculated from RFU [-] measured from cell suspension with established correlation equation y = 4837.0 x + 8697.6 (linear fitting, R^2^ 0.96) from *E. coli* BL21 T-resp. expression system in M9 media (10 g/L glucose) cultivated at 35 °C over 24 h in the shake flask setup described above.

### Data fitting, parameter optimization, and modelling

RNAT expression systems can be classified as so-called single-stage processes [50]. In contrast to conventional two-stage processes, where biomass formation and product synthesis are temporally separated, biomass growth and product formation occur simultaneously, resulting in competing metabolic fluxes. Additionally, temperature plays a dual role in these systems, acting both as an induction trigger and as a key process parameter influencing cellular physiology.

The representation of cultivation data based solely on maximum values or endpoint measurements may lead to misleading interpretations. This is particularly critical during the early phases of cultivation, where mathematical equations can introduce artifacts due to initial conditions and model assumptions.

A frequently underestimated challenge arises in the context of substrate-based data analysis. While experimental quantification typically considers the total substrate consumption, this does not necessarily reflect the fraction of substrate that is metabolically available for specific pathways. Instead, substrates are distributed across competing cellular processes, leading to trade-offs between measured substrate uptake and its effective utilization within distinct metabolic routes. Additional complexities related to scaling and system behavior are discussed in section Challenges in scaling RNAT-based expression systems from flask to bioreactor, Fig. 7. These considerations raise the question of how experimental data can be presented in a manner that is transparent, comparable, and biologically meaningful, while adequately capturing the influence of temperature, minimizing the impact of outliers, and accounting for potential mathematical artifacts.

In a first analysis step experimental data of biomass, product, acetate, and glucose time courses were fitted using a four-parameter logarithmic fit adapted from Henkel and colleagues described in Eq. 1 [51]. OriginPro, Version 2023 (OriginLab Corporation, Northampton, MA, USA) was used for data plotting. The specific growth rate (*µ*) in Eq. 2, and the specific production rate (*q*_P_) in Eq. 3, were calculated between time intervals (t_i_ and t_i−1_) and normalized on the average biomass *c*_X, av_ allowing reduced influence of mathematical artifacts, especially occurring in the beginning of the cultivation.

Calculation of yields is commonly performed between the start and the end of cultivation time or the start and the maxima. Here, we define the calculation of yields (Eqs. 4–8) between the start of cultivation (‘) and the timepoint of substrate depletion (*, glucose concentration < 0.5 g/L).

1 $$\:\mathrm{y}={\mathrm{y}}_{0}+\frac{\mathrm{a}}{1+{\left(\frac{\mathrm{x}}{{\mathrm{x}}_{0}}\right)}^{\mathrm{b}}}$$

2 $$\:\mu\:=\:\frac{\frac{{c}_{\mathrm{X},\mathrm{i}}-{c}_{\mathrm{X},\mathrm{i}-1}}{{\mathrm{t}}_{\mathrm{i}}-{\mathrm{t}}_{\mathrm{i}-1}}}{{c}_{\mathrm{X},\mathrm{a}\mathrm{v}}}$$

3 $$\:{q}_{\mathrm{P}}=\:\frac{\frac{{c}_{\mathrm{P},\mathrm{i}}-{c}_{\mathrm{P},\mathrm{i}-1}}{{\mathrm{t}}_{\mathrm{i}}-{\mathrm{t}}_{\mathrm{i}-1}}}{{c}_{\mathrm{X},\mathrm{a}\mathrm{v}}}$$

4 $$\:{Y}_{\mathrm{X}/\mathrm{S}}=\:\frac{{c}_{\mathrm{X},\mathrm{S}\mathrm{*}}-{c}_{\mathrm{X},\mathrm{S}^{\prime\:}}}{{c}_{\mathrm{S}^{\prime\:}}-{c}_{\mathrm{S}\mathrm{*}}}$$

5 $$\:{Y}_{\mathrm{P}/\mathrm{S}}=\:\frac{{c}_{\mathrm{P},\mathrm{S}\mathrm{*}}-{c}_{\mathrm{P},\mathrm{S}^{\prime\:}}}{{c}_{\mathrm{S}^{\prime\:}}-{c}_{\mathrm{S}\mathrm{*}}}$$

6 $$\:{Y}_{\mathrm{P}/\mathrm{X}}=\:\frac{{c}_{\mathrm{P},\mathrm{S}\mathrm{*}}-{c}_{\mathrm{P},\mathrm{S}^{\prime}}}{{c}_{\mathrm{X},\mathrm{S}\mathrm{*}}-{c}_{\mathrm{X},\mathrm{S}^{\prime\:}}}$$

7 $$\:{Y}_{\mathrm{A}\mathrm{c}/\mathrm{S}}=\:\frac{{c}_{\mathrm{A}\mathrm{c},\mathrm{S}\mathrm{*}}-{c}_{\mathrm{A}\mathrm{c},\mathrm{S}^{\prime\:}}}{{c}_{\mathrm{S}^{\prime\:}}-{c}_{\mathrm{S}\mathrm{*}}}$$

8 $$\:{Y}_{\mathrm{P},\mathrm{m}\mathrm{a}\mathrm{x}/\mathrm{A}\mathrm{c}}=\:\frac{{c}_{\mathrm{P},\mathrm{m}\mathrm{a}\mathrm{x}}-{c}_{\mathrm{P}^{\prime\:}}}{{c}_{\mathrm{A}\mathrm{c},\mathrm{P}\mathrm{m}\mathrm{a}\mathrm{x}}-{c}_{\mathrm{A}\mathrm{c},\mathrm{P}^{\prime\:}}}$$

*Y* = yield [g/g].

*µ* = growth rate [h^-1^].

*q*_P_ = specific production rate [g_P_ / (g_X_ ∙ h)]

*c*_X_ = biomass concentration [g/L].

*c*_S_ = glucose concentration [g/L].

*c*_P_ = EmGFP concentration [g/L].

*c*_Ac_ = acetate concentration [g/L].

*c*_X, av_ = average biomass concentration [g/L], approximated in intervals by $\:\frac{{c}_{\mathrm{X},\mathrm{i}}\:+\:{c}_{\mathrm{X},\mathrm{i}-1}}{2}$.

t = cultivation time [h].

i = indices of cultivation time points.

*‘* = start concentration (time point).

* = end concentration (time point).

max = maximum concentration.

MATLAB 2025b (The MathWorks, Natick, MA, USA) was used to describe the temperature dependency of specific average EmGFP production rates (*q*_P, av_) by applying the empirical model by Ratkowsky and team, adapted by Zwietering, Noll, and colleagues [31, 52, 53] presented in Eq. 9. Parameter optimization was performed using the Nelder-Mead algorithm, executed via the fmincon and fminsearch functions. The process minimized the least-squares error between the simulated and measured data.

9 $$\:\mathrm{u}\left(\mathrm{T}\right)={\left[\mathrm{b}\cdot\:\left(\mathrm{T}-{\mathrm{T}}_{\mathrm{m}\mathrm{i}\mathrm{n}}\right)\right]}^{2}\cdot\:\{1-{\mathrm{e}}^{\mathrm{c}\cdot\:(\mathrm{T}-{\mathrm{T}}_{\mathrm{m}\mathrm{a}\mathrm{x}})}\}$$

## Results and discussion

Characterization of temperature-responsive (T-resp.), derepressed (control), as well as no plasmid (NP) constructs were conducted in M9 media in shake flasks with a starting glucose concentration of 10 g/L. The controls used in this study were chosen based on comprehensive prior characterization, ensuring well-defined structural and regulatory impacts of the introduced point mutation (A14-U25 to G14-C25 [33]). Randomly modified sequences were omitted, because of the unclear change in conformation potentially leading to artifacts or effects of different origin. To investigate the translation control efficiency using RNA thermometers (RNAT) in a plasmid-based system, a temperature range (25–40 °C) was investigated. Besides recombinant protein production, bioprocess indicators including yields and specific rates, altered carbon partitioning, and (un-)specific metabolic effects were analyzed and applied to a temperature-dependent model. To the best of our knowledge, this study presents the first quantitative analysis of recombinant protein expression controlled by an RNAT system.

### Time courses and process performance parameters

**Fig. 2:**
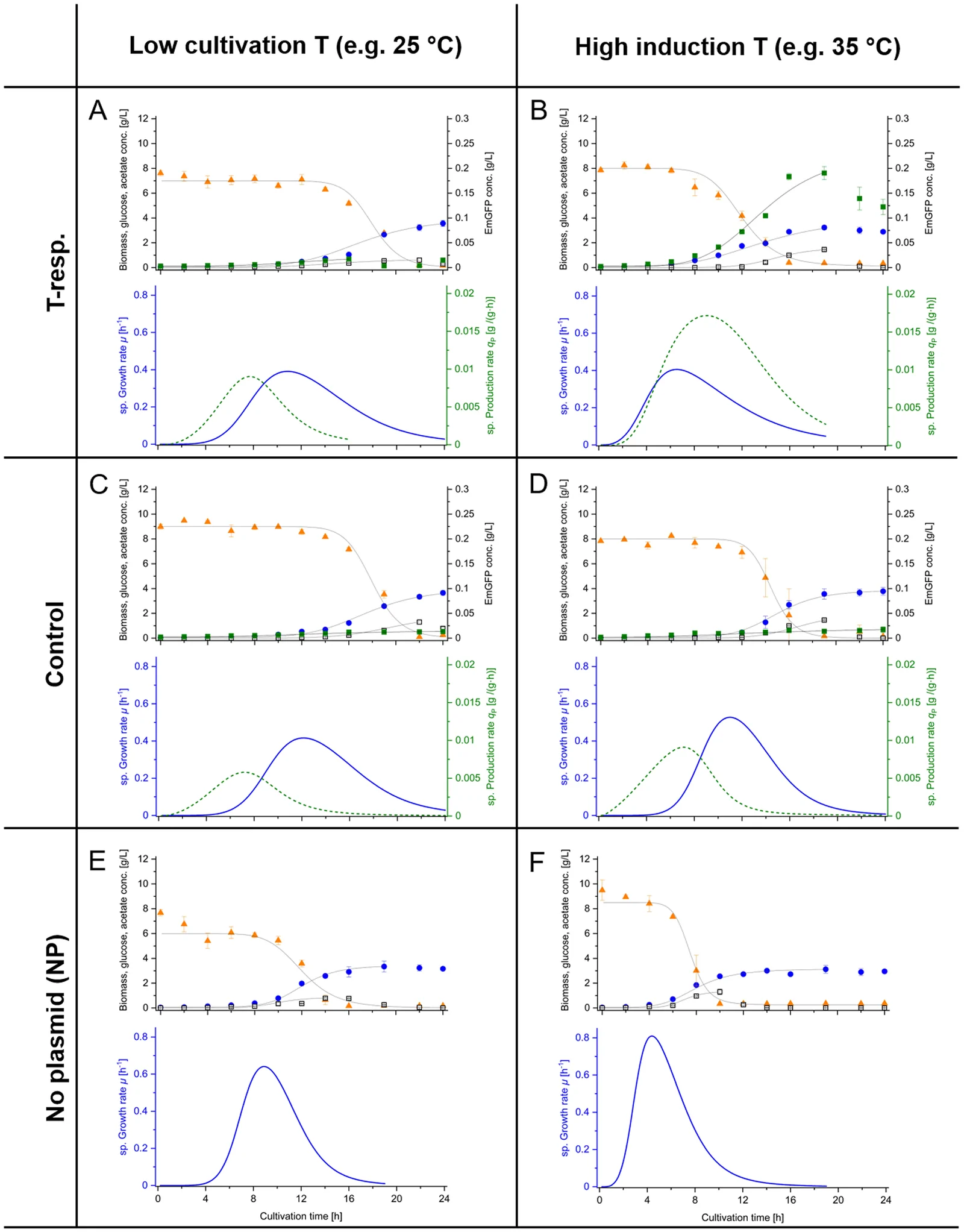
Time course of glucose concentration *c*_S_ [g/L] (orange triangles), biomass concentration *c*_X_ [g/L] (blue circles), acetate concentration *c*_Ac_ [g/L] (unfilled black squares), EmGFP concentration *c*_P_ [g/L] (filled green squares), specific growth rate *µ* [h^− 1^] (blue line), and specific production rate *q*_P_ [g/ (g∙h)] (green dotted line) of shake flask cultivation *E. coli* BL21 temperature responsive (T-resp.) element (**A & B**), non-temperature-responsive (control) (**C & D**) and no plasmid (NP) (**E & F**) at two temperatures (e.g. 25 °C low cultivation temperature and e.g. 35 °C induction temperature) recorded over 24 h of cultivation

Figure 2 highlights two representative temperatures corresponding to non-induced (low cultivation temperature i.e. 25 °C, Fig. 2A, C, E) and induced (higher cultivation temperature i.e. 35 °C, Fig. 2B, D, F) as time course curves of biomass concentration *c*_X_ [g/L], glucose concentration *c*_S_ [g/L], acetate concentration *c*_Ac_ [g/L], and EmGFP concentration *c*_P_ [g/L] over 24 h. Furthermore, the specific (sp.) growth rate *µ* as well as the specific (sp.) production rate *q*_P_ are presented. Raw data of all experiments conducted will be provided upon request.

Cultivation of *E. coli* BL21 T-resp. showed similar maximum biomass conc. of 3.56 ± 0.24 g/L after 24 h of cultivation at 25 °C (Fig. 2A) and after 19 h of 3.23 ± 0.16 g/L for cultivation at 35 °C (Fig. 2B). Glucose depletion (< 0.50 g/L) occurred after 22 h for cultivation at 25 °C and after 16 h when cultivated at 35 °C. Maximum EmGFP conc. was observed after 19 h of cultivation at 35 °C (0.191 ± 0.013 g/L) compared to a maximum of 0.017 ± 0.001 g/L at 25 °C after 16 h of cultivation. The effect of temperature influencing rather protein expression (approx. 10-fold increase), than biomass formation reveals first hints on an active translation regulation by RNAT. The derepressed control showed overall slower glucose consumption and decelerated EmGFP production compared to the T-resp. construct at 25 °C. Glucose was fully consumed after 22 h (< 0.50 g/L) at 25 °C (Fig. 2C) resulting in a maximum biomass conc. of 3.65 ± 0.19 g/L and a maximum of 0.014 ± 0.001 g/L EmGFP after 24 h. At 35 °C (Fig. 2D), resulting in maximum biomass conc. of 3.78 ± 0.31 g/L and 0.017 ± 0.006 g/L EmGFP after 24 h. Glucose was depleted after 19 h (< 0.50 g/L) of cultivation. Interestingly, for the control similar protein expression levels, as well as biomass formation were observed, indicating minor temperature-responsive behavior.

*E. coli* BL21 NP showed similar growth behavior at 25 °C (Fig. 2E) and 35 °C (Fig. 2F). Cultivation at 35 °C resulted in glucose depletion after 10 h (< 0.50 g/L) and a maximum biomass conc. of 3.12 ± 0.31 g/L after 19 h. For lower cultivation temperature, at 25 °C glucose depletion occurred after 14 h, and a maximum biomass conc. of 3.34 ± 0.43 g/L after 19 h of cultivation.

For further evaluation, the specific growth rate *µ* and specific production rate *q*_P_ presented in Fig. 2 were calculated based on logarithmic fits derived from biomass and product time courses (Eqs. 1, 2, 3). The T-resp. construct showed similar maximum sp. growth rate *µ*_max_ of 0.39 h^− 1^ after 10.8 h at 25 °C and 0.41 h^− 1^ after 6.6 h at 35 °C. The control construct as well as the NP did show increased *µ*_max_ at 35 °C (0.53 h^− 1^ after 11.0 h, 0.81 h^− 1^ after 4.4 h^− 1^) compared to 25 °C (0.42 h^− 1^ after 12.1 h, 0.64 h^− 1^ after 8.9 h^− 1^). The NP construct showed the fastest glucose depletion and the highest *µ*_max_, when comparing all three constructs, most likely due to the lack of metabolic burden. Furthermore, maximum specific production rate *q*_P, max_ of the control construct showed similar expression at 25 °C (0.007 g/(g∙h) after 8.0 h) and 35 °C (0.009 g/(g∙h) after 7.1 h), evaluating it as a suitable control construct in this study. While *q*_P, max_ of the T-resp. construct shows similar results to the control at 25 °C (0.009 g/(g∙h) after 7.7 h), *q*_P, max_ almost doubles at 35 °C (0.017 g/(g∙h) after 8.6 h). Supporting data of *µ*_max_ and *q*_P, max_ is presented in the supplementary information, Table S1.

Hence, data provide evidence of enhanced biosynthesis of EmGFP compared to biomass production. The titers quantified in this study are within the described range 0.05–0.30 g/L for constitutive promoters with medium strength used in batch cultivation, based on previous reports [11, 54, 55]. Optimal growth temperature of *E. coli* is described at approx. 37 °C [56], depending on the strain and metabolic burden. Therefore, it is expected that the investigated expression systems show overall decelerated activity at less than 35 °C. Above 39 °C, temperature-induced damage of biomass can be expected. However, lower cultivation temperatures are described to favor correct protein folding and functional biomass formation as well as less degradation [50, 57]. The applied RNA thermometer sequence (fourU) is reported to be regulatory active in the temperature range of approx. 30–42 °C [25, 33, 36, 44], which overlaps with the optimal growth condition of the host organism. Increased translation at higher temperatures may impose a higher metabolic burden on the host system, however, it may also promote faster mRNA turnover rates due to unfolded RNA structures, which can affect both growth and recombinant protein production. Furthermore, previous studies have reported enhanced production levels solely due to the presence of the RNAT sequence itself. However, the underlying molecular mechanisms responsible for these beneficial regulatory and stabilizing effects are not yet fully understood. Correspondingly, the study of Noll and colleagues describes such observations for a functional ROSE-like RNAT in *Pseudomonas putida* KT2440 [31]. Their data reveal interference and partial competition of the expression system and the host system, resulting in shifted temperature optima for growth and product formation. Thus, thermo-induction affects not only the translation process, but also causes various overlapping biochemical alterations in the entire expression system.

### Temperature-dependent model for the specific production rate

**Fig. 3:**
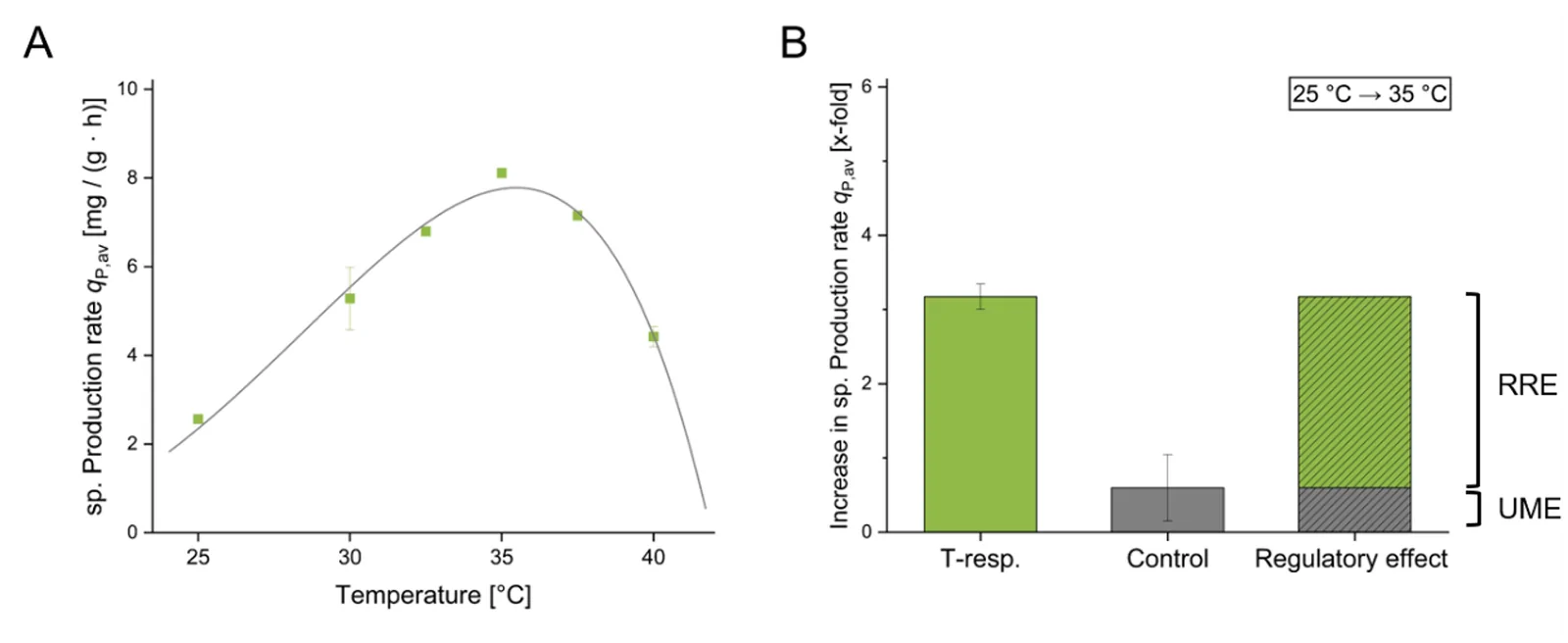
Temperature-dependent specific (sp.) protein production rate as well as regulatory effects under conditions of increased temperature. **A**) Average specific (sp.) production rate *q*_P, av_ [mg/(g ∙ h)] calculated of shake flask cultivation of T-resp. construct over 24 h (green squares) plotted against cultivation temperature from 25–40 °C. Grey line shows an empirical temperature model ([52] adapted by [53, 58], Eq. 9) fitting experimental data. **B**) Visualization of RNAT regulatory effect (RRE, green shaded) and unspecific metabolic effect (UME, grey shaded) as x-fold of the T-resp. (green) and the control (grey) construct when comparing *q*_P, av_ at 25 °C and 35 °C

In depth analysis of the specific production rate *q*_P, av_ [mg/(g ∙ h)] obtained from experimental data over tested temperature range (25–40 °C) revealed maxima at 35 °C for the T-resp. (8.11 ± 0.11 mg/(g ∙ h)) and at 30 °C for the control (3.29 ± 0.39 mg/(g ∙ h)). The control showed temperature-independent translation across tested range of 25–40 °C with *q*_P, av_ remaining approx. 5.00 mg/(g ∙ h). Furthermore, a temperature-dependent model for *q*_P_ based on experimental data describing *q*_P, av_ as a function of temperature (25–40 °C) was used for the prediction of optimal cultivation temperature. The empirical model by Ratkowsky and team (described in section Data fitting, parameter optimization, and modelling, Eq. 9) of *q*_P, av_ vs. temperature was parametrized using the experimentally derived specific production rates [52] adapted by Zwietering, Noll, and colleagues [53, 58]. Based on the model estimate peak *q*_P, av_ appears at 7.78 mg/(g ∙ h) and 35.51 °C (Fig. 3A). Data is provided in the supplementary information, Table S3. Compared to literature gained *q*_P_ are on the lower end of the described range between approx. 5.0–25.0 mg/(g ∙ h) for constitutive promoters, potentially reflecting the low-to-medium promoter strength [11, 54, 55].

### RNAT regulatory and unspecific metabolic regulatory effect

Based on the evaluated efficiency parameters, 30 °C was pre-selected as temperature setpoint for growth, and 35 °C as the setpoint for protein production. The increase in *Y*_P/X_ when comparing fitted data of cultivations at 30 °C and 35 °C for the T-resp. construct was approx. 3.0-fold. The approx. 0.9-fold change of *Y*_P/X_ of the respective control experiment can be exemplarily defined as the unspecific metabolic effect (UME), while an approx. 2.1-fold change can be determined as the RNAT regulatory effect (RRE) (see supplementary information, Fig. S3A). Here, for reasons of simplicity, we do not assume synergistic or additive effects. To further investigate the temperature-dependent regulation, an extended comparison between 25 °C and 35 °C was performed. An approx. 9.0-fold change of RRE and 1.2-fold change of UME of *Y*_P/X_ could be determined by comparing cultivations at 25 °C and 35 °C (see supplementary information, Fig. S3B). Increased RRE can be observed under conditions of sustained growth, combined with low EmGFP production of the T-resp. system at 25 °C.

When focusing on experimental data of *q*_P, av_ an increase of approx. 2.6-fold change of RRE and 0.6-fold change of UME of the average specific production rate *q*_P, av_ in mg/(g ∙ h) comparing 25 °C and 35 °C can be determined as illustrated in Fig. 3B. Findings are consistent with previous reported studies [25, 32, 33, 44]. An even stronger effect, most likely due to the evaluation of obtained *q*_P, max_ data (in comparison to the *q*_P, av_ presented in this study) was reported by Noll and team, revealing an approx. 9.3-fold change of RRE in the investigated glycolipid production system using *P. putida* KT2440 [44]. Furthermore, it is stated that a strong increase of *q*_P_ between two evaluated temperatures highlights the potential of future temperature induction strategies for future bioprocess applications, which is also supported by the findings of the present study. A systematic comparison of different RNATs was beyond the scope of the present study, which focused on the quantitative characterization of the selected fourU RNAT. However, such a comparison would be required to assess differences in performance and transferability across RNAT systems.

### Evaluation of altered carbon partitioning

**Fig. 4:**
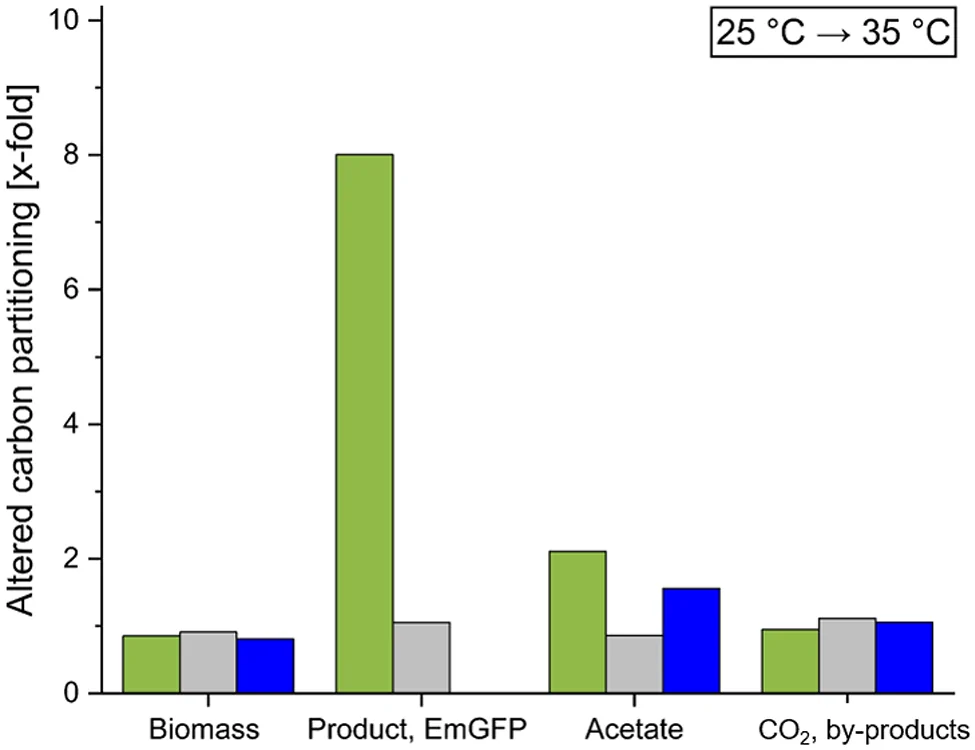
Altered carbon partitioning as fold change comparing temperatures 25 °C and 35 °C. Evaluation of biomass, product (EmGFP), acetate, and CO_2_ (and other byproducts, not considered) of the T-resp. (green), control (grey), and no plasmid (NP) (blue) construct

One major advantage of using RNAT in general as gene expression control is the potential of fine-tuning substrate flow into product rather than biomass without relying on established strategies such as inducers or auxotrophy. Altered carbon partitioning was evaluated for two selected temperatures (25 °C and 35 °C) of the T-resp., control, and NP construct as presented in Fig. 4. Supporting data are provided in the supplementary information Table S4. Data of biomass conc., EmGFP conc., and by-product, i.e. acetate conc. per substrate were balanced, respectively, and compared within one construct at 25 °C and 35 °C as well as in relation to all tested constructs. Subsequently, C-mole fractions were calculated and results are presented as an increase in altered carbon partitioning as fold change comparing temperatures 25 °C and 35 °C (Fig. 4).

All three tested constructs show similar increases when evaluating carbon partitioning into biomass (0.81–0.91 -fold change) and CO_2_ (and other by-products, not considered) (0.95–1.12 -fold change) when comparing 25 °C and 35 °C.

The T-resp. construct exhibits an increased altered carbon partitioning of 2.11-fold change (6.0% and 12.7% of total carbon) into acetate compared to 0.86-fold change of the control construct (12.7% and 10.9% of total carbon), and 1.56-fold change of the NP construct (8.4% and 13.1% of total carbon).

Interestingly, while the control construct reveals similar carbon partitioning into product (approx. 1-fold change), the T-resp. construct reveals a highly increased partitioning into product by an 8-fold change (0.3% and 2.2% of total carbon). Results confirm that altered carbon partitioning is linked to the regulatory mechanism of the applied RNAT, favoring product formation at 35 °C. Although the significance of this effect (precise control of substrate flow by temperature changes) requires further investigation, the present results demonstrate fundamental steps toward future decoupling conditions using functional RNAT as „translation enhancers“ compared to derepressed control structures. Consistent with these findings, previous studies demonstrated that the promoter choice, RBS composition, and mRNA secondary structure can substantially influence translation efficiency, mRNA stability, and ultimately protein expression levels [59, 60]. Future trials can build on the presented reference system, enabling induction approaches similar to the approach evaluated by Noll and team [44]. Moreover, a proposed advantage of RNAT is the reversible induction mechanism, which has been intensively reviewed [6, 35, 36, 41]. Combining advanced feeding strategies, genetic algorithms, and molecular process control elements such as RNATs could significantly enhance bioprocess performance for recombinant protein production. Ultimately, process efficiency may be improved to a level that exceeds established industrial production systems and enables the competitiveness of future precision fermentation applications.

### Accumulated yields as process indicators

**Fig. 5:**
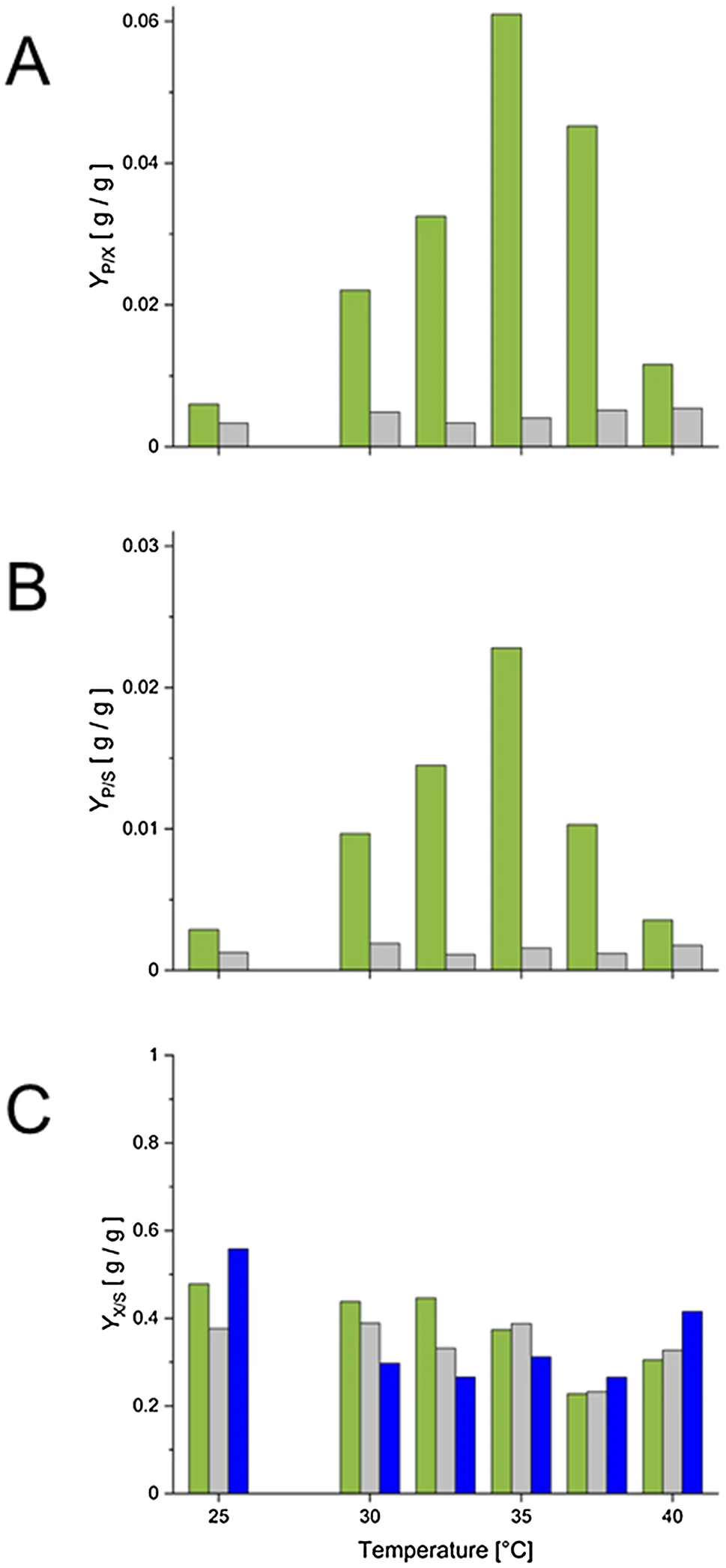
Comparison of substrate-, biomass-, and product-yields for T-resp. system compared to controls. **A**) *Y*_P/X_ [g_EmGFP_ / g_Biomass_] of T-resp. (green) and the control (grey) construct over temperature range 25–40 °C. **B**) *Y*_P/S_ [g_EmGFP_ / g_Glucose_] of T-resp. (green) and the control (grey) construct over temperature range 25–40 °C. **C**) *Y*_X/S_ [g_Biomass_ / g_Glucose_] of T-resp. (green), the control (grey), and no plasmid (NP) construct (blue) over temperature range 25–40 °C

In the following, accumulated yields *Y* [g/g] are evaluated to characterize process performance and to identify potential optima for growth, and protein production, as well as altered carbon partitioning. Supporting data of Fig. 5 are provided in the supplementary information, Table S2. Calculations were performed according to the section Data fitting, parameter optimization, and modelling (Eqs. 4, 5, 6).

Figure 5A presents product-per-biomass yields (*Y*_P/X_) of the T-resp and the control construct over a temperature range of 25–40 °C. Whereas *Y*_P/X_ appears almost independent of temperature for the control construct, a maximum can be identified at 37.5 °C (0.0054 g/g). In contrast, the maximum for the T-resp. construct appears at 35 °C (0.0610 g/g), which corresponds to a total accumulated increase of approx. 10-fold compared to the control.

In Fig. 5B product-per-substrate yields (*Y*_P/S_) of the T-resp. and control systems over a temperature range 25–40 °C are presented. Interestingly, the T-resp. construct showed a maxima at 35 °C (0.0228 g/g), similar to observations for *Y*_P/X_. As previously evaluated, the control is minimally affected by temperature changes (maxima at 30 °C, 0.0019 g/g), whereas the T-resp. construct shows temperature dependency.

Compared to *Y*_P/X_ between 25 °C and 35 °C, *Y*_P/S_ was increased similarly by approx. 10-fold. As a result, *Y*_P/X_ and *Y*_P/S_ both show temperature optima towards 35 °C for the T-resp. construct. Although the observed increase appears substantial, it reflects cumulative yield formation over the entire cultivation period (24 h) rather than an instantaneous regulatory effect. At earlier cultivation stages, differences between the T-resp. and control constructs were less pronounced. This behavior results from the temporal separation of biomass accumulation and subsequent product formation, supporting the potential of RNAT-mediated regulation to establish distinct growth and production phases. Subsequently, further evaluation was conducted comparing substrate conversion as presented in Fig. 5C, showing biomass-per-substrate yields (*Y*_X/S_) of the T-resp. system, the control, and the NP construct over the tested temperature range 25–40 °C.

For biomass-per-substrate yield (*Y*_X/S_), the T-resp. construct showed a distinct minimum at 37 °C, which was also reflected by the corresponding control strains, suggesting a temperature-dependent increase in cellular maintenance demand rather than a construct-specific regulatory effect. Such partial competition between the expression system and the host has previously been discussed in the section Time courses and process performance parameters. At lower temperatures, *Y*_X/S_ was higher for the T-resp. construct, as inhibited recombinant protein synthesis (RNAT blocks translation), enabled preferential partitioning of substrate carbon toward biomass formation. In contrast, the control constitutively distributed resources between biomass and product formation across all temperatures. The NP exhibited increased *Y*_X/S_ values at the temperature extremes of 25 °C and 40 °C, suggesting altered substrate utilization under suboptimal growth conditions, whereas lower yields at intermediate temperatures (30–37 °C) may reflect increased metabolic activity and associated maintenance requirements. These overall observations are in agreement with described *Y*_X/S_ in literature for *E. coli* where a range from 0.3 to 0.6 for *Y*_X/S_ is stated [10, 56]. Furthermore, the increased yields observed at temperatures below the optimal growth temperature support the previously stated hypothesis of increased functional biomass formation and improved cell population viability. Interestingly, *Y*_X/S_ did not differ substantially between NP and the strains containing a plasmid, despite the expectation that plasmid maintenance and expression would impose an additional metabolic burden. This suggests that the overall efficiency of substrate conversion into biomass remained largely unaffected under the investigated conditions. It is worth noting that this discrepancy may also arise from the mathematical definition of *Y*_X/S_ applied in this study (see also Eq. 4). In contrast, the growth rate profiles (see also Fig. 2) provide a more detailed and time-resolved assessment of cellular physiology, revealing that NP exhibited a higher *µ*_max_ and more rapid glucose consumption.

### Acetate formation and by-product related yields

**Fig. 6:**
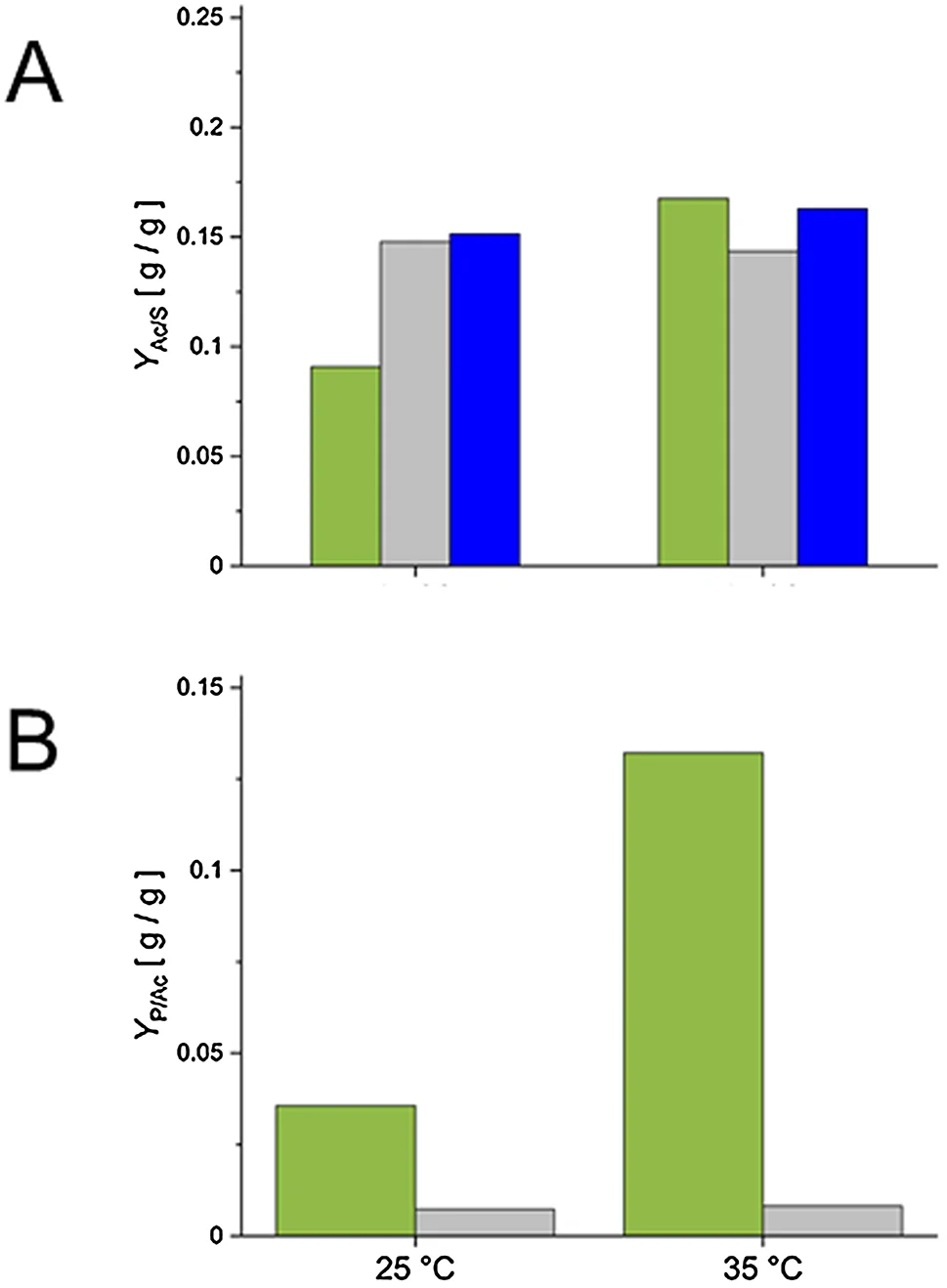
Comparison of byproduct-yields for T-resp. system compared to controls. **A**) *Y*_Ac/S_ [g_Acetate_ / g_Glucose_] of T-resp. (green), the control (grey), and no plasmid (NP) (blue) construct at 25 °C and 35 °C. **B**) *Y*_P/Ac_ [g_EmGFP, max_ / g_Acetate_] of T-resp. (green), and the control (grey) construct at 25 °C and 35 °C

To investigate a broader range of efficiency parameters and potential effects of RNAT-mediated regulation on the overall metabolism and expression system, by-product yields were also analyzed. Acetate formation in *E. coli* strains is a key indicator for overfeeding and, therefore, exceeding respiratory capacities. As an undesired by-product it is named as one of the main drawbacks of using *E. coli* as a production host, if not addressed properly by genetic modifications [9, 13, 18]. Recent metabolic engineering approaches established acetate-reduced and negative strains for industrial application. Figure 6 presents acetate-per-substrate (*Y*_Ac/S_) and product-per-acetate yields (*Y*_P/Ac_). Calculations were performed according to the section Data fitting, parameter optimization, and modelling (Eq. 7, and 8).

In Fig. 6A by-product (acetate)-per-substrate yields (*Y*_Ac/S_) of the T-resp., control, and NP construct at 25 °C and 35 °C are presented. *Y*_Ac/S_ of NP appears similar at 25 °C and 35 °C (0.151 g/g, 0.163 g/g) due to no plasmid burden i.e., no burden of recombinant protein production or selection pressure. Likewise, the control shows similar *Y*_Ac/S_ at 25 °C and at 35 °C (0.148 g/g, 0.143 g/g). The T-resp. construct shows an increase in *Y*_Ac/S_ at 35 °C (0.168 g/g) compared to 25 °C (0.091 g/g). This effect may result from a combination of temperature-dependent factors, such as increased glucose uptake rates resulting in overflow metabolism, or the metabolic burden imposed by the constitutive promoter together with the regulatory element (RNAT blocks translation). Such conditions may induce elevated cellular stress, leading to increased absolute acetate formation and consequently higher *Y*_Ac/S_ values. Moreover, acetate accumulation is directly linked to the central carbon metabolism and occurs when glucose uptake exceeds the respiratory capacity of the cells. Hence, it enables direct assessment of metabolic responses associated with RNAT-controlled expression systems. However, *E. coli* BL21 generally exhibits lower acetate formation compared to K-12-derived strains, which has been associated with its slower glucose uptake rate and consequently more efficient utilization of the tricarboxylic acid (TCA) cycle.

In order to evaluate process efficiency in depth *Y*_P/Ac_ (i.e. yield calculated from maximum product conc. over corresponding time point and acetate conc.) was evaluated as an additional parameter shown in Fig. 6B. *Y*_P/Ac_ of the control construct showed similar levels resulting in 0.007 g/g at 25 °C and 0.008 g/g at 35 °C. The T-resp. construct resulted in higher *Y*_P/Ac_ at both 25 °C (0.036 g/g) and 35 °C (0.132 g/g). Consequently, the expression system appears to be highly active under these conditions, especially when considering elevated metabolic host activity. However, further characterization and evaluation is needed to attribute a potential regulatory effect to the applied regulatory RNA element.

### Challenges in scaling RNAT-based expression systems from flask to bioreactor

**Fig. 7:**
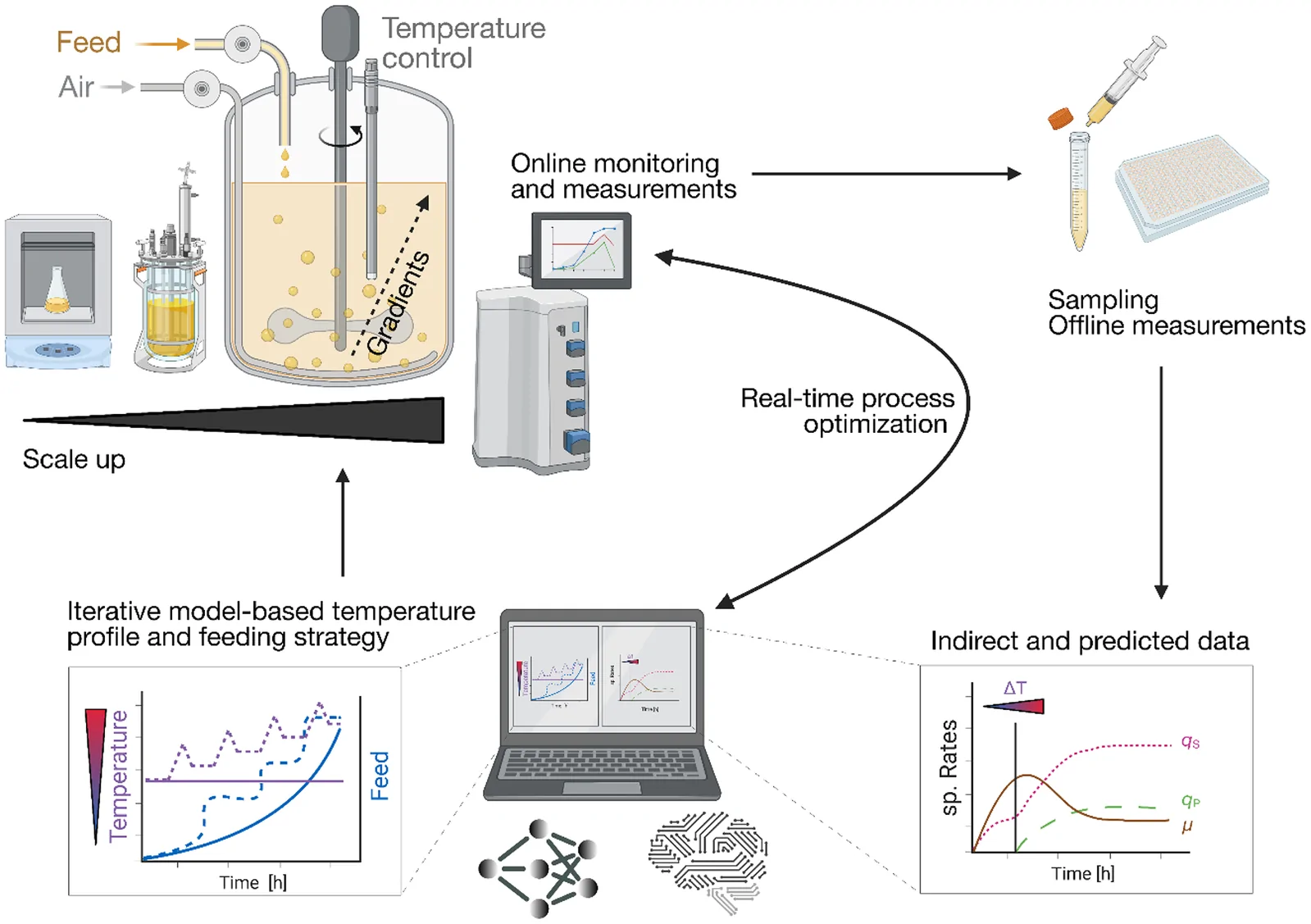
Model-assisted scaling of RNAT-based expression systems from flask to large-scale bioreactor. Created in BioRender. Peternell, C. (2026) https://BioRender.com/mra23hg

The presented results demonstrate the potential of RNAT as a tool for decoupling biomass formation from recombinant protein production. However, the transfer of this approach from laboratory scale (e.g. shake flasks) into a controlled large-scale bioreactor setup introduces additional layers of complexity and therefore requires real-time and iterative model-assisted optimization as illustrated in Fig. 7.

A central challenge arises from temperature acting as both a regulatory trigger and a key player in cellular physiology [36, 44, 50]. RNATs as regulatory elements are highly sensitive to temperature changes, capable of responding to alterations within a narrow temperature range [6]. At lower temperatures, enzymatic activity and growth rates are reduced, and the RNAT is predominantly in the “off” state, resulting in a low abundance of recombinant protein production (see also Fig. 1) [6, 23, 25, 36]. In contrast, increasing temperature generally enhances enzymatic activity and growth rate up to an optimum, while simultaneously cellular maintenance energy demand is elevated. In the context of RNAT-controlled systems, this creates a non-linear interplay between growth and recombinant production since activation of the 4U RNAT (“on”) promotes recombinant protein synthesis [25]. Hence, this imposes the common trade-offs of single-state systems, like an additional metabolic burden that potentially reduces biomass formation efficiency. Consequently, growth and production are not fully decoupled and are limited by competing cellular demands, such as ribosomal capacity and energy metabolism, and substrate allocation.

In contrast to shake flask cultivations, large-scale bioprocesses are inherently heterogeneous due to longer mixing times, resulting in gradients in temperature, substrate availability, and dissolved oxygen [57, 61, 62]. Although temperature measurement is well established at industrial scale, multi-point online sensor systems may be required to accurately monitor temperature gradients in large-scale bioreactors, where pronounced thermal heterogeneities can arise due to cooling being primarily applied through the vessel walls. Since RNAT-based regulation directly depends on temperature, gradients may lead to non-uniform induction (levels) across the culture, potentially resulting in heterogeneous product quality and quantity per cell. Moreover, besides the effect on metabolic conversions, e.g. by increasing enzymatic reaction times, temperature shifts can influence additional physical and physiological parameters, including oxygen solubility, mass transfer rates, protein folding kinetics, stress responses, and mRNA stability [50, 63]. Rapid heating times are required for fast simultaneous induction. However, these are expected to increase with reactor scale due to changes in the surface-to-volume ratio. In addition, the available surface area per unit volume decreases, thereby limiting heat transfer efficiency. Consequently, less surface is available for heat exchange per unit volume, resulting in slower temperature equilibration in larger reactors increasing the potential occurrence of gradients [50, 62]. The multifactorial dependency highlights that RNAT-based systems introduce a dynamic temperature response that differs fundamentally from conventional chemically induced processes. Hence, this poses a significant challenge for fed-batch processes, where substrate feeding strategies are typically designed based on relatively stable physiological parameters [50, 57, 63, 64]. These effects are further complicated by temperature-dependent overall metabolism and substrate uptake rates. A mismatch between feed rate and actual substrate demand can lead to overflow metabolism, such as acetate formation in *E. coli*, or alternatively to substrate limitation and reduced productivity [63].

The observed coupling between temperature, specific growth rate (*µ*), specific substrate uptake rate (*q*_S_), and specific production rate (*q*_P_) suggests that conventional feeding strategies may be insufficient. To address these challenges, the development of predictive models that integrate temperature-dependent kinetics, metabolic flux redistribution, and regulatory effects will be essential [51, 58, 65]. The initial datasets required for model development are obtained through design of experiments and experimental evaluations, including both online, e.g. exhaust gas analysis, and offline measurements, e.g. absorbance assays. Subsequently, hybrid approaches combining mechanistic modeling with data-driven methods, such as machine learning or artificial neural networks (ANN), may offer promising approaches. Mears and colleagues propose suitable strategies to establish adaptive control schemes for fed-batch processes [57]. Soft sensors and real-time product monitoring could further support the implementation of adaptive control in response to dynamically changing process conditions [66, 67]. From an industrial perspective, RNAT-based regulation offers clear advantages, including a chemical inducer-free system and the potential for reversible induction by temperature. In industrial applications, the RNAT-based expression system could function via autoinduction by generated metabolic heat as proposed by Noll and colleagues [44].

As such, the present work aims to establish the necessary basis for reference datasets and system-specific parameters required for the development of model-based temperature profiles and feeding strategies. These models will enable a more reliable process understanding and supporting systematic process development and optimization of RNAT-based expression systems. Future work should therefore focus on validating the observed decoupling effects in controlled bioreactor systems and developing advanced control frameworks that can fully exploit the potential of temperature-responsive gene regulation.

## Conclusion

This work demonstrates the potential of RNA thermometers (RNATs) as temperature-responsive molecular process control elements for precision fermentation. Using Emerald green fluorescent protein (EmGFP) as a reporter, RNAT-mediated translational regulation was quantitatively characterized and related to recombinant protein production, yield formation, and carbon allocation within the bioprocess.

The absolute EmGFP concentration increased approx. 10-fold change at the induction temperature of 35 °C (0.191 ± 0.013 g/L) compared to 25 °C, indicating a strong temperature-dependent regulatory response. Functional RNAT-mediated regulation was demonstrated by a gradual increase in specific production rate (*q*_P_), reaching a maximum at 35 °C (*q*_P, av_ 8.11 ± 0.11 mg/(g∙h) and *q*_P, max_ 17.0 mg/(g∙h)). Furthermore, the 2.6-fold change in *q*_P, av_ between 25 °C and 35 °C supported a distinct RNAT-mediated regulatory contribution in addition to temperature-dependent physiological effects. Nevertheless, temperature elevation also affects host physiology through changes in metabolic activity, oxygen availability, and other cellular processes, complicating the direct attribution of production changes solely to functional RNAT elements.

Evaluation of carbon partitioning revealed that elevated induction temperature partially altered substrate carbon towards product formation while carbon allocation into biomass remained largely unchanged. Notably, the temperature-responsive construct showed an approx. increase of an 8-fold change in carbon partitioning toward product formation compared to the control, as reflected by higher *Y*_P/S_ values. These findings provide a basis for future process optimization strategies aimed at strengthening the decoupling of growth and production phases through temperature-shift approaches. Future studies combining transcriptomic and metabolomic analyses may further elucidate the mechanisms underlying RNAT-mediated regulation and identify additional targets for process optimization. In addition, tailored feeding strategies and model-based optimization approaches may support predictive control of these complex temperature-regulated production systems. Future work may further expand this concept through the integration of RNATs with different promoter systems or by combining heat- and cold-responsive RNATs to enable more sophisticated control of growth and recombinant protein production.

Taken together, the expression platform, in combination with temperature modulation, enables altered carbon partitioning at the process level from biomass formation towards product synthesis, thereby improving substrate conversion efficiency and overall process performance. This capability is particularly relevant in the context of precision fermentation, where efficient carbon utilization and controlled product formation are key determinants of economic feasibility. In this regard, molecular bioprocess control exploiting RNATs represents a promising strategy for next-generation precision fermentation processes, particularly for the sustainable and economically viable production of bulk food proteins and other recombinant products.

## Supplementary Information

Below is the link to the electronic supplementary material.

Supplementary Material 1

## Data Availability

The datasets used and/or analyzed during the current study are available from the corresponding author on reasonable request.

## Abbreviations

4U: Four uracil residues
ANN: Artificial neural network
conc.: Concentration
*E. coli*: *Escherichia coli*
EmGFP: Emerald green fluorescent protein
MPC: Molecular process control
mRNA: Messenger RNA
NP: No plasmid construct
*P*: Product [g/L]
PF: Precision fermentation
*q*_P_: Specific production rate [g/(g·h)]
*q*_S_: Specific substrate uptake rate
RBS: Ribosome binding site
RNAT: RNA thermometer
*S*: Substrate [g/L]
SD: Shine-Dalgarno sequence
T: Temperature [°C]
T-resp.: Temperature responsive
t: Time [h]
UTR: Untranslated region
wt: Wildtype
*X*: Biomass [g/L]
*Y*: Yield [g/g]
*µ*: Specific growth rate [h^− 1^]

## References

[1] Beck F, Noll P, Schweiggert-Weisz U, Henkel M. Scientific and technological challenges of recombinant egg protein production. BMC Biotechnol. 2025;25:65. 10.1186/s12896-025-01002-3. PMC12224593 40604696

[2] Nielsen MB, Meyer AS, Arnau J. The Next Food Revolution Is Here: Recombinant Microbial Production of Milk and Egg Proteins by Precision Fermentation. Annual Rev Food Sci Technol. 2024;15:173–87. 10.1146/annurev-food-072023-034256. 38134386

[3] El Wali M, Rahimpour Golroudbary S, Kraslawski A, Tuomisto HL. Transition to cellular agriculture reduces agriculture land use and greenhouse gas emissions but increases demand for critical materials. Commun Earth Environ. 2024;5:61. 10.1038/s43247-024-01227-8.

[4] Chai KF, Ng KR, Samarasiri M, Chen WN. Precision fermentation to advance fungal food fermentations. Curr Opin Food Sci. 2022;47:100881. 10.1016/j.cofs.2022.100881.

[5] Dupuis JH, Cheung LKY, Newman L, Dee DR, Yada RY. Precision cellular agriculture: The future role of recombinantly expressed protein as food. Comp Rev Food Sci Food Safe. 2023;22:882–912. 10.1111/1541-4337.13094. 36546356

[6] Sharma P, Mondal K, Kumar S, Tamang S, Najar IN, Das S, et al. RNA thermometers in bacteria: Role in thermoregulation. Biochim et Biophys Acta (BBA) - Gene Regul Mech. 2022;1865:194871. 10.1016/j.bbagrm.2022.194871. 36041664

[7] Teng TS, Chin YL, Chai KF, Chen WN. Fermentation for future food systems: Precision fermentation can complement the scope and applications of traditional fermentation. EMBO Rep. 2021;22:e52680. 10.15252/embr.202152680. PMC8097352 33908143

[8] Terefe NS. Recent developments in fermentation technology: toward the next revolution in food production. Food Engineering Innovations Across the Food Supply Chain [Internet]. Elsevier; 2022 [cited 2023 Nov 1]. pp. 89–106. 10.1016/B978-0-12-821292-9.00026-1.

[9] De Mey M, De Maeseneire S, Soetaert W, Vandamme E. Minimizing acetate formation in E. coli fermentations. J Ind Microbiol Biotechnol. 2007;34:689–700. 10.1007/s10295-007-0244-2. 17668256

[10] Marisch K, Bayer K, Scharl T, Mairhofer J, Krempl PM, Hummel K et al. A comparative analysis of industrial *Escherichia coli* K–12 and B strains in high-glucose batch cultivations on process-, transcriptome- and proteome level. Chatterji D, editor. PLoS ONE. 2013;8:e70516. 10.1371/journal.pone.0070516 PMC3738542 23950949

[11] Shiloach J, Fass R, Growing. *E. coli* to high cell density—A historical perspective on method development. Biotechnol Adv. 2005;23:345–57. 10.1016/j.biotechadv.2005.04.004. 15899573

[12] Xu B, Jahic M, Enfors S-O. Modeling of Overflow Metabolism in Batch and Fed-Batch Cultures of *Escherichia coli*. Biotechnol Prog. 1999;15:81–90. 10.1021/bp9801087. 9933517

[13] Bernal V, Castaño-Cerezo S, Cánovas M. Acetate metabolism regulation in *Escherichia coli*: carbon overflow, pathogenicity, and beyond. Appl Microbiol Biotechnol. 2016;100:8985–9001. 10.1007/s00253-016-7832-x. 27645299

[14] De Groot DH, Lischke J, Muolo R, Planqué R, Bruggeman FJ, Teusink B. The common message of constraint-based optimization approaches: overflow metabolism is caused by two growth-limiting constraints. Cell Mol Life Sci. 2020;77:441–53. 10.1007/s00018-019-03380-2. PMC7010627 31758233

[15] Pfeiffer T, Morley A. An evolutionary perspective on the Crabtree effect. Front Mol Biosci [Internet]. 2014. 10.3389/fmolb.2014.00017. [cited 2026 Mar 2];1. PMC4429655 25988158

[16] Thierie J, Penninckx M, Crabtree, effect. Encyclopedia of industrial biotechnology [Internet]. 1st ed. Wiley; 2010 [cited 2026 Mar 2]. pp. 1–18. 10.1002/9780470054581.eib243.

[17] Wojtczak L. The Crabtree effect: a new look at the old problem. Acta Biochim Pol. 1996;43:361–8. 10.18388/abp.1996_4505. 8862181

[18] Wolfe AJ. The Acetate Switch. Microbiol Mol Biol Rev. 2005;69:12–50. 10.1128/MMBR.69.1.12-50.2005. PMC1082793 15755952

[19] Hansen LH, Knudsen S, Sørensen SJ. The Effect of the *lacY* Gene on the Induction of IPTG Inducible Promoters, Studied in *Escherichia coli* and *Pseudomonas fluorescens*. Curr Microbiol. 1998;36:341–7. 10.1007/s002849900320. 9608745

[20] Jobe A, Bourgeois S. lac repressor-operator interaction. J Mol Biol. 1972;69:397–408. 10.1016/0022-2836(72)90253-7. 4562709

[21] Neubauer P, Hofmann K, Holst O, Mattiasson B, Kruschke P. Maximizing the expression of a recombinant gene in *Escherichia coli* by manipulation of induction time using lactose as inducer. Appl Microbiol Biotechnol [Internet]. 1992 [cited 2025 Nov 25];36. 10.1007/BF00172185 1369364

[22] Treinen C, Peternell C, Noll P, Magosch O, Hausmann R, Henkel M. Molecular process control for industrial biotechnology. Trends Biotechnol. 2025. 10.1016/j.tibtech.2025.04.003. S0167779925001301. 40335343

[23] Narberhaus F, Waldminghaus T, Chowdhury S. RNA thermometers. FEMS Microbiol Rev. 2006;30:3–16. 10.1111/j.1574-6976.2005.004.x. 16438677

[24] Nshogozabahizi JC, Aubrey KL, Ross JA, Thakor N. Applications and limitations of regulatory RNA elements in synthetic biology and biotechnology. J Appl Microbiol. 2019;127:968–84. 10.1111/jam.14270. 30927378

[25] Waldminghaus T, Heidrich N, Brantl S, Narberhaus F. FourU: a novel type of RNA thermometer in *Salmonella*. Mol Microbiol. 2007;65:413–24. 10.1111/j.1365-2958.2007.05794.x. 17630972

[26] Altuvia S, Kornitzer D, Teff D, Oppenheim AB. Alternative mRNA structures of the *cIII* gene of bacteriophage λ determine the rate of its translation initiation. J Mol Biol. 1989;210:265–80. 10.1016/0022-2836(89)90329-X. 2532257

[27] Hoe NP, Goguen JD. Temperature sensing in *Yersinia pestis*: translation of the LcrF activator protein is thermally regulated. J Bacteriol. 1993;175:7901–9. 10.1128/jb.175.24.7901-7909.1993. PMC206968 7504666

[28] Nocker A, Krstulovic N-P, Perret X, Narberhaus F. ROSE elements occur in disparate rhizobia and are functionally interchangeable between species. Arch Microbiol. 2001;176:44–51. 10.1007/s002030100294. 11479702

[29] Waldminghaus T, Fippinger A, Alfsmann J, Narberhaus F. RNA thermometers are common in α- and γ-proteobacteria. Biol Chem. 2005;386:1279–86. 10.1515/BC.2005.145. 16336122

[30] Grosso-Becerra MV, Croda-García G, Merino E, Servín-González L, Mojica-Espinosa R, Soberón-Chávez G. Regulation of *Pseudomonas aeruginosa* virulence factors by two novel RNA thermometers. Proc Natl Acad Sci USA. 2014;111:15562–7. 10.1073/pnas.1402536111. PMC4217398 25313031

[31] Noll P, Treinen C, Müller S, Senkalla S, Lilge L, Hausmann R, et al. Evaluating temperature-induced regulation of a ROSE-like RNA-thermometer for heterologous rhamnolipid production in *Pseudomonas putida* KT2440. AMB Expr. 2019;9:154. 10.1186/s13568-019-0883-5. PMC6761213 31555921

[32] Meyer S, Carlson PD, Lucks JB. Characterizing the Structure–Function Relationship of a Naturally Occurring RNA Thermometer. Biochemistry. 2017;56:6629–38. 10.1021/acs.biochem.7b01170. PMC5807002 29172455

[33] Rinnenthal J, Klinkert B, Narberhaus F, Schwalbe H. Modulation of the stability of the *Salmonella* fourU-type RNA thermometer. Nucleic Acids Res. 2011;39:8258–70. 10.1093/nar/gkr314. PMC3185406 21727085

[34] Rinnenthal J, Klinkert B, Narberhaus F, Schwalbe H. Direct observation of the temperature-induced melting process of the *Salmonella* fourU RNA thermometer at base-pair resolution. Nucleic Acids Res. 2010;38:3834–47. 10.1093/nar/gkq124. PMC2887971 20211842

[35] Chowdhury S, Maris C, Allain FH-T, Narberhaus F. Molecular basis for temperature sensing by an RNA thermometer. EMBO J. 2006;25:2487–97. 10.1038/sj.emboj.7601128. PMC1478195 16710302

[36] Kortmann J, Narberhaus F, Bacterial. RNA thermometers: molecular zippers and switches. Nat Rev Microbiol. 2012;10:255–65. 10.1038/nrmicro2730. 22421878

[37] Kammerdiener EK, Garcia SK, Bales MK, Klingeman DM, Guss AM, Giannone RJ, et al. Multilayered regulation by RNA thermometers enables precise control of Cas9 expression in *E. coli*. Nucleic Acids Res. 2026;54:gkaf1495. 10.1093/nar/gkaf1495. PMC12802932 41533573

[38] Hoynes-O’Connor A, Hinman K, Kirchner L, Moon TS. De novo design of heat-repressible RNA thermosensors in *E. coli*. Nucleic Acids Res. 2015;43:6166–79. 10.1093/nar/gkv499. PMC4499127 25979263

[39] Neupert J, Karcher D, Bock R. Design of simple synthetic RNA thermometers for temperature-controlled gene expression in *Escherichia coli*. Nucleic Acids Res. 2008;36:e124–124. 10.1093/nar/gkn545. PMC2577334 18753148

[40] Neupert J, Bock R. Designing and using synthetic RNA thermometers for temperature-controlled gene expression in bacteria. Nat Protoc. 2009;4:1262–73. 10.1038/nprot.2009.112. 19680240

[41] Sen S, Apurva D, Satija R, Siegal D, Murray RM. Design of a Toolbox of RNA Thermometers. ACS Synth Biol. 2017;6:1461–70. 10.1021/acssynbio.6b00301. 28437108

[42] Sharts DM, Almanza MT, Banks AV, Castellanos AM, Hernandez O, Lopez CG. Robo-Therm, a pipeline to RNA Thermometer discovery and validation. RNA. 2024. 10.1261/rna.079980.124. rna.079980.124. PMC11182007 38565243

[43] Jia H, Sun X, Sun H, Li C, Wang Y, Feng X, et al. Intelligent Microbial Heat-Regulating Engine (IMHeRE) for Improved Thermo-Robustness and Efficiency of Bioconversion. ACS Synth Biol. 2016;5:312–20. 10.1021/acssynbio.5b00158. 26793993

[44] Noll P, Treinen C, Müller S, Lilge L, Hausmann R, Henkel M. Exploiting RNA thermometer-driven molecular bioprocess control as a concept for heterologous rhamnolipid production. Sci Rep. 2021;11:14802. 10.1038/s41598-021-94400-4. PMC8292423 34285304

[45] Peternell C, Noll P, Brümmer-Rolf A, Henkel M. Making GFP count: a validated framework for absolute protein quantification in precision fermentation. Appl Microbiol Biotechnol. 2026;110:56. 10.1007/s00253-026-13734-z. PMC12876113 41639258

[46] Anderson JC, Dueber JE, Leguia M, Wu GC, Goler JA, Arkin AP, et al. BglBricks: A flexible standard for biological part assembly. J Biol Eng. 2010;4:1. 10.1186/1754-1611-4-1. PMC2822740 20205762

[47] Bertani G. Studies on lysogenesis I. The mode of phage liberation by lysogenic *Escherichia coli*. J Bacteriol. 1951;62:293–300. 10.1128/jb.62.3.293-300.1951. PMC386127 14888646

[48] Dulbecco R, Vogt M. Plaque formation and isolation of pure lines with poliomyelitis viruses. J Exp Med. 1954;99:167–82. 10.1084/jem.99.2.167. PMC2180341 13130792

[49] Henkel M, Zwick M, Beuker J, Willenbacher J, Baumann S, Oswald F, et al. Teaching bioprocess engineering to undergraduates: Multidisciplinary hands-on training in a one‐week practical course. Biochem Mol Biol Educ. 2015;43:189–202. 10.1002/bmb.20860. PMC6680437 25820495

[50] Hartline CJ, Schmitz AC, Han Y, Zhang F. Dynamic control in metabolic engineering: Theories, tools, and applications. Metab Eng. 2021;63:126–40. 10.1016/j.ymben.2020.08.015. PMC8015268 32927059

[51] Henkel M, Schmidberger A, Vogelbacher M, Kühnert C, Beuker J, Bernard T, et al. Kinetic modeling of rhamnolipid production by Pseudomonas aeruginosa PAO1 including cell density-dependent regulation. Appl Microbiol Biotechnol. 2014;98:7013–25. 10.1007/s00253-014-5750-3. 24770383

[52] Ratkowsky DA, Olley J, McMeekin TA, Ball A. Relationship between temperature and growth rate of bacterial cultures. J Bacteriol. 1982;149:1–5. 10.1128/jb.149.1.1-5.1982. PMC216584 7054139

[53] Zwietering MH, De Koos JT, Hasenack BE, De Witt JC, Van ’T, Riet K. Modeling of bacterial growth as a function of temperature. Appl Environ Microbiol. 1991;57:1094–101. 10.1128/aem.57.4.1094-1101.1991. PMC182851 2059034

[54] Baneyx F. Recombinant protein expression in *Escherichia coli*. Curr Opin Biotechnol. 1999;10:411–21. 10.1016/S0958-1669(99)00003-8. 10508629

[55] Rosano GL, Ceccarelli EA. Recombinant protein expression in *Escherichia coli*: advances and challenges. Front Microbiol [Internet]. 2014. 10.3389/fmicb.2014.00172. [cited 2024 Feb 4];5. PMC4029002 24860555

[56] Shuler ML, Kargi F, DeLisa M. Bioprocess engineering: basic concepts. Third ed. Boston Columbus Indianapolis New York San Francisco Amsterdam Cape Town Dubai London Madrid Milan Munich Paris Montreal Toronto Delhi Mexico City São Paulo Sydney Hong Kong Singapore Taipei Tokyo: Prentice Hall; 2017.

[57] Mears L, Stocks SM, Sin G, Gernaey KV. A review of control strategies for manipulating the feed rate in fed-batch fermentation processes. J Biotechnol. 2017;245:34–46. 10.1016/j.jbiotec.2017.01.008. 28179156

[58] Noll P, Lilge L, Hausmann R, Henkel M. Modeling and Exploiting Microbial Temperature Response. Processes. 2020;8:121. 10.3390/pr8010121.

[59] Kosuri S, Goodman DB, Cambray G, Mutalik VK, Gao Y, Arkin AP, et al. Composability of regulatory sequences controlling transcription and translation in *Escherichia coli*. Proc Natl Acad Sci USA. 2013;110:14024–9. 10.1073/pnas.1301301110. PMC3752251 23924614

[60] Salis HM, Mirsky EA, Voigt CA. Automated design of synthetic ribosome binding sites to control protein expression. Nat Biotechnol. 2009;27:946–50. 10.1038/nbt.1568. PMC2782888 19801975

[61] Burg JM, Cooper CB, Ye Z, Reed BR, Moreb EA, Lynch MD. Large-scale bioprocess competitiveness: the potential of dynamic metabolic control in two-stage fermentations. Curr Opin Chem Eng. 2016;14:121–36. 10.1016/j.coche.2016.09.008.

[62] Alford JS. Bioprocess control: Advances and challenges. Comput Chem Eng. 2006;30:1464–75. 10.1016/j.compchemeng.2006.05.039.

[63] Wechselberger P, Sagmeister P, Engelking H, Schmidt T, Wenger J, Herwig C. Efficient feeding profile optimization for recombinant protein production using physiological information. Bioprocess Biosyst Eng. 2012;35:1637–49. 10.1007/s00449-012-0754-9. PMC3470689 22740334

[64] Jenzsch M, Gnoth S, Beck M, Kleinschmidt M, Simutis R, Lübbert A. Open-loop control of the biomass concentration within the growth phase of recombinant protein production processes. J Biotechnol. 2006;127:84–94. 10.1016/j.jbiotec.2006.06.004. 16962679

[65] Wendering P, Nikoloski Z. Model-driven insights into the effects of temperature on metabolism. Biotechnol Adv. 2023;67:108203. 10.1016/j.biotechadv.2023.108203. 37348662

[66] Luttmann R, Bracewell DG, Cornelissen G, Gernaey KV, Glassey J, Hass VC, et al. Soft sensors in bioprocessing: A status report and recommendations. Biotechnol J. 2012;7:1040–8. 10.1002/biot.201100506. 22489000

[67] Sagmeister P, Wechselberger P, Jazini M, Meitz A, Langemann T, Herwig C. Soft sensor assisted dynamic bioprocess control: Efficient tools for bioprocess development. Chem Eng Sci. 2013;96:190–8. 10.1016/j.ces.2013.02.069.

