# Supplementary material for: RNA thermometers as temperature-dependent tools for enhanced recombinant protein production in precision fermentation
Source: Microb Cell Fact. 2026 Oct 13;25:211. doi: 10.1186/s12934-026-03143-2 (PMC13670657; doi:10.1186/s12934-026-03143-2)
Supplement: Supplementary file 1 [file 12934_2026_3143_MOESM1_ESM.docx]

Supporting Information

**RNA thermometers as temperature-dependent tools for enhanced recombinant protein production in precision fermentation**

Christina Peternell, Philipp Noll, and Marius Henkel*

*Corresponding author: Prof. Dr.-Ing. habil. Marius Henkel

Address: Gregor-Mendel-St. 4, 85354 Freising, Germany

Email: [marius.henkel@tum.de](mailto:marius.henkel@tum.de)

RNA thermometer structure prediction

**Fig. S1** Predicted RNA thermometer structure (Waldminghaus et al. 2007 doi: 10.1111/j.1365-2958.2007.05794.x, Rinnenthal et al. 2010 doi: 10.1093/nar/gkq124 and 2011 doi: 10.1093/nar/gkr314, Meyer et al. 2017 doi: 10.1021/acs.biochem.7b01170, Noll et al. 2021 doi: 10.1038/s41598-021-94400-4, Sharma et al. 2022 doi: 10.1016/j.bbagrm.2022.194871) **A)** FourU wildtype (T-responsive construct), and **B)** FourU derepressed (G14A-C25U point mutation, control construct). Created with *mFold 4.1* (Zuker 2003 doi: 10.1093/nar/gkg595)

Plasmid maps of expression systems used in this study

**Fig. S2** Plasmid map of **A)** pRSET_J23108_4U(wt)_EmGFP (3321 bp) abbreviated as temperature responsive (T-resp. construct) construct, and **B)** pRSET_J23108_4U(dest, G14A-C25U)_EmGFP (3321 bp) abbreviated as control construct. Created with *Benchling.com*

Regulatory effect of RNA thermometer

**Fig. S3** Evaluation of RNA thermometer regulatory effect (RRE) and unspecific metabolic effect (UME) as **A)** x-fold increase of *Y*_P/X_ of T-resp. and the control construct compared at 30 °C and 35 °C, and **B)** x-fold increase of *Y*_P/X_ of T-resp. and the control construct compared at 25 °C and 35 °C.

Evaluation of specific process rates

**Table S1** overview of evaluated maximum specific production rate *q*_P,max_ and maximum specific growth rate *μ*_max_ of the T-resp., control, and no plasmid (NP) construct at 25 °C and 35 °C

|  | **T-resp.** | | **Control** | | **NP** | |
| --- | --- | --- | --- | --- | --- | --- |
| **Temperature [°C]** | ***q*_P,max_ [g/(g∙h)]** | ***μ*_max_ [h^-1^]** | ***q*_P,max_ [g/(g∙h)]** | ***μ*_max_ [h^-1^]** | ***q*_P,max_ [g/(g∙h)]** | ***μ*_max_ [h^-1^]** |
| **25.0** | 0.01 | 0.39 | 0.01 | 0.42 | - | 0.64 |
| **35.0** | 0.02 | 0.41 | 0.01 | 0.53 | - | 0.81 |

Evaluation of yield coefficients

**Table S2** overview of evaluated yields of the T-resp., control, and no plasmid (NP) construct over temperature range 25 °C - 40 °C

| **T-resp.** | | | | | | |
| --- | --- | --- | --- | --- | --- | --- |
| **Temperature [°C]** | ***Y* _X/S_ [g / g]** | ***Y* _P/S_ [g / g]** | ***Y* _P/X_ [g / g]** | ***Y* _Ac/S_ [g / g]** | ***Y* _P/Ac_ [g / g]** |  |
| **25.0** | 0.478 | 0.003 | 0.006 | 0.091 | 0.036 |  |
| **30.0** | 0.438 | 0.010 | 0.022 | - | - |  |
| **32.5** | 0.446 | 0.015 | 0.033 | - | - |  |
| **35.0** | 0.374 | 0.023 | 0.061 | 0.168 | 0.132 |  |
| **37.5** | 0.228 | 0.010 | 0.045 | - | - |  |
| **40.0** | 0.305 | 0.004 | 0.012 | - | - |  |
| **Control** | | | | | | |
| **Temperature [°C]** | ***Y* _X/S_ [g / g]** | ***Y* _P/S_ [g / g]** | ***Y* _P/X_ [g / g]** | ***Y* _Ac/S_ [g / g]** | ***Y* _P/Ac_ [g / g]** |  |
| **25.0** | 0.376 | 0.001 | 0.003 | 0.148 | 0.007 |  |
| **30.0** | 0.389 | 0.002 | 0.005 | - | - |  |
| **32.5** | 0.332 | 0.001 | 0.003 | - | - |  |
| **35.0** | 0.388 | 0.002 | 0.004 | 0.143 | 0.008 |  |
| **37.5** | 0.232 | 0.001 | 0.005 | - | - |  |
| **40.0** | 0.327 | 0.002 | 0.005 | - | - |  |

| **NP** | | | | | | |
| --- | --- | --- | --- | --- | --- | --- |
| **Temperature [°C]** | ***Y* _X/S_ [g / g]** | ***Y* _P/S_ [g / g]** | ***Y* _P/X_ [g / g]** | ***Y* _Ac/S_ [g / g]** | ***Y* _P/Ac_ [g / g]** |  |
| **25.0** | 0.558 | - | - | 0.151 | - |  |
| **30.0** | 0.297 | - | - | - | - |  |
| **32.5** | 0.266 | - | - | - | - |  |
| **35.0** | 0.312 | - | - | 0.163 | - |  |
| **37.5** | 0.265 | - | - | - | - |  |
| **40.0** | 0.415 | - | - | - | - |  |

Average specific production rate *q*_P,av_ prediction over the temperature range 25-40 °C

**Table S3** Average specific production rate (*q*_P,av experimental_) determined experimentally and fitted (*q*_P,av fitted_) of the T-resp. construct using descriptive temperature model (Ratkowsky et al. 1982 doi: 10.1128/jb.149.1.1-5.1982, Zwietering et al. 1991 doi: 10.1128/aem.57.4.1094-1101.1991, Noll et al. 2020 doi: 10.3390/pr8010121 and 2021 doi: 10.1038/s41598-021-94400-4)

| **Temperature [°C]** | ***q*_P,av experimental_**  **[mg / (g ∙ h)]** | ***q*_P,av fitted_**  **[mg / (g ∙ h)]** |
| --- | --- | --- |
| **25.0** | 2.56 ± 0.11 | 2.35 |
| **30.0** | 5.28 ± 0.70 | 5.54 |
| **32.5** | 6.80 ± 0.03 | 6.97 |
| **35.0** | 8.11 ± 0.11 | 7.75 |
| **37.5** | 7.15 ± 0.09 | 7.23 |
| **40.0** | 4.42 ± 0.22 | 4.45 |

Altered carbon partitioning

**Table S4** Evaluation of altered carbon partitioning (compared at 25 °C and 35 °C and) presented as -x-fold change of the T-resp., control, and no plasmid (NP) construct

| **Altered carbon partitioning  [x-fold change]** | **T-resp.** | **Control** | **NP** |
| --- | --- | --- | --- |
| **Biomass** | 0.85 | 0.91 | 0.81 |
| **Product, EmGFP** | 8.01 | 1.05 | - |
| **Acetate** | 2.11 | 0.86 | 1.56 |
| **CO_2_ and other by-products** | 0.95 | 1.12 | 1.06 |
